# Causal associations between dietary antioxidant vitamin intake and lung cancer: A Mendelian randomization study

**DOI:** 10.3389/fnut.2022.965911

**Published:** 2022-09-02

**Authors:** Hang Zhao, Xiaolin Jin

**Affiliations:** ^1^Peking University China-Japan Friendship School of Clinical Medicine, Beijing, China; ^2^Department of Urology, China-Japan Friendship Hospital, Beijing, China; ^3^Department of International Physical Examination Center, The First Affiliated Hospital of China Medical University, Shengyang, China

**Keywords:** antioxidants, vitamin intake, lung cancer, Mendelian randomization, causation

## Abstract

**Background:**

Oxidative stress is currently considered to be closely related to the occurrence of respiratory tumors, especially lung cancer. Many observational studies have shown that increased antioxidant intake can reduce the risk of lung cancer, but the results are still controversial. Therefore, we performed a two-sample Mendelian randomized (MR) analysis to clarify the causal relationship between antioxidant vitamins and lung cancer.

**Methods:**

To assess the causal effect of dietary antioxidant vitamin intake on lung cancer, we conducted a two-sample MR analysis and we extracted single-nucleotide polymorphisms (SNPs) that are associated with antioxidants from genome-wide association studies (GWASs) of the UK biobank. We gathered summary data for lung cancer from the International Lung Cancer Consortium (ILCCO), including 11,348 cases and 15,861 controls, and applied the inverse-variance weighted (IVW) method as the primary MR analysis, and performed a sensitivity analysis to verify the results.

**Results:**

The results showed that higher dietary retinol intake was causally associated with lung cancer overall [odds ratio (*OR*) = 1.844, 95% *CI*, 1.359–2.502, *p* = 0.00009], squamous cell lung cancer (*OR* = 2.162, 95% *CI*, 1.117–4.183, *p* = 0.022), and lung adenocarcinoma (*OR* = 1.706, 95% *CI*, 1.084–2.685, *p* = 0.021). Additionally, carotene was positively correlated with lung adenocarcinoma (*OR* = 1.510, 95% *CI*, 1.002–2.276, *p* = 0.049). However, there was a non-significant relationship between the intake of other dietary antioxidants (vitamin C and vitamin E) and lung cancer.

**Conclusion:**

Our research showed that dietary retinol intake has an adverse impact on lung cancer, and carotene might increase the risk of adenocarcinoma. This highlights the importance of revealing the underlying mechanisms of dietary antioxidant vitamins in lung cancer and delivers an important health message that dietary antioxidant vitamin intake may not be necessary for the prevention of lung cancer. It also provides a basis for future research.

## Introduction

Lung cancer is one of the most frequent cancers in the world (1). In recent years, it has remained the primary cause of cancer death (2). According to a cancer statistical study, the number of new lung cancer incidences was 2.2 million, and the number of new deaths was 1.8 million in 2020. In terms of incidence and mortality, the most common form of cancer among men is lung cancer, while it ranks third among women. The incidence rate and mortality rate of men are roughly two times those of women (2). Lifestyle and environmental factors can significantly influence the risk of lung cancer.

Smoking, body mass index (BMI), asbestos, and air pollution were regarded as traditional risk factors for lung cancer (3, 4). The molecular genetics of lung cancer are also important, and the development of malignant lung tumors seems to be due to the multistage molecular pathogenesis, as well as the accumulation and combination of genetic factors and abnormalities (5). A large amount of evidence and studies show that mutations of the Kirsten rat sarcoma viral oncogene (KRAS), epidermal growth factor receptor (EGFR), vascular endothelial growth factor (VEGF), and P53 play an important role in the occurrence of lung cancer (6–8). EGFR mutations are closely related to lung cancer in never smokers (9), whereas mutations in KRAS are strongly associated with lung cancer in people who are smoking (10). Patients who have never smoked have also been observed to have specific mutations of the KRAS oncogene with more guanine-adenosine transitions in codons 12 and 13, and anaplastic lymphoma kinase (ALK) gene rearrangements occur more frequently in non-smokers (11, 12). The mutation difference shows that in the never smoking population, the development path of lung cancer is independent of tobacco smoke, and there are still limitations between smoking and lung cancer risk (13, 14). However, at least one selected effective tobacco-control measure was implemented in 136 countries, covering 65% of the world's population (15). Approximately 67% of all lung cancer deaths worldwide can be attributed to smoking (2).

Recently, studies suggested that antioxidant vitamins or minerals are associated with the risk of cancer, such as gastric cardia cancer, prostate cancer, and lung cancer (16–20). However, the conclusions of these studies were inconsistent. For lung cancer, it is now widely believed that oxidative stress plays important roles in tumor growth and development (21, 22). Many studies concluded that diet-derived antioxidants, such as retinol, were associated with a reduced risk of lung cancer (16, 23). For instance, a 10.6-years cohort study found that dietary vitamin E intake was associated with a lower risk of lung cancer (24). Several meta-analysis studies had the same results, suggesting that higher dietary antioxidant vitamins (vitamin A, carotene, and vitamin C) intake could reduce the risk of lung cancer (25, 26). Zhu et al. performed a dose-response meta-analysis and found that for every extra 2 mg/day of vitamin E intake, lung cancer risks decreased by 5% (27). However, it is difficult to draw firm conclusions. Narita et al. found that the dietary consumption of retinol increased the risk of lung cancer in men in the Japan Public Health Center-based Prospective Study. However, they failed to find the effects of the intake of other antioxidant vitamins on lung cancer (28). Many other studies have not found an influence of antioxidant vitamin intake on lung cancer (29). Similarly, mineral intake is controversial for the occurrence of lung cancer (30). An analysis of 482,875 subjects in the National Institute of Health (NIH)-American Association of Retired Persons Diet and Health study was conducted by Mahabir et al. They observed an increased risk of dietary Mg in all subjects, with the study population being men who were current smokers (31). However, Muka et al. revealed no evidence of an association between magnesium, calcium, selenium, and copper intake and lung cancer risk (32). These controversial results may be attributed to the bias of confounders. In addition, conventional observational studies have methodological limitations, and the inverse causal relationship between antioxidant vitamin intake and lung cancer should also be considered.

Mendelian randomization (MR) analysis is a special type of instrumental variable analysis that can reduce the potential bias from residual confounding and reverse causations (33). In MR analysis, we use single nucleotide polymorphisms (SNPs) as instrumental variables (IVs) for detecting and quantifying casual associations between exposures and outcomes (34). At present, a number of observational studies and trials are currently investigating the association between vitamins and cancer risk (35, 36), but the findings remain inconclusive. Many MR studies have focused on the potential causal association between vitamins and cancer, such as circulating vitamins B, C, D, and E (37–41). For lung cancer, it has been shown that serum retinol may be a risk factor for lung cancer (42), while the other circulating antioxidant vitamins have no effect on lung cancer risk (37, 39, 42). However, due to the unique bioaccessibility of antioxidants, it has not been determined whether dietary antioxidants affect the incidence of lung cancer. Thus, to estimate the causal association between dietary antioxidant vitamin intake and lung cancer risk, we carried out a two-sample MR analysis.

## Methods

### Study design

We performed a two-sample MR analysis based on a large-scale genome-wide association study (GWAS) for recognized antioxidant vitamins [vitamin A (retinol), carotene, vitamin C, and vitamin E] (43, 44), lung cancer, squamous cell lung cancer, and lung adenocarcinoma. The design of our study is shown in Supplementary Figures 1, 2. There are three principal assumptions of MR analysis: (1) a close relationship should exist between exposure and genetic variants; (2) genetic variants should be independent of confounders; and (3) genetic variants can only affect the outcome through this exposure.

### Instrumental variables

We searched the MR database for GWAS data on dietary retinol, carotene, vitamin C, and vitamin E intake. We selected the genetic variants with genome-wide significance (*p* < 5 × 10^−6^) as IVs. Furthermore, to ensure the independence of IVs, we set *r*^2^ < 0.001 and clump distance >10,000 kb as the linkage disequilibrium (LD) threshold. If there was more than one GWAS, we selected the largest one. Thus, 48 IVs associated with retinol, carotene, vitamin C, and vitamin E were obtained from the data of Integrative Epidemiology Unit (IEU) analysis of the UK Biobank (the number of European participants ranged from 62,991 to 64,979). We collected 9 GWAS-identified retinol-associated (UK Biobank Data-Field 100018) single-nucleotide polymorphisms, 16 SNPs associated with carotene (UK Biobank Data-Field 100019), 11 SNPs associated with vitamin C (UK Biobank Data-Field 100015), and 12 SNPs associated with vitamin E (UK Biobank Data-Field 100025). These data are all from the food and beverage intake of the participants. Furthermore, we searched the PhenoScanner database, which contains large-scale data of GWAS, to check the associations between the included SNPs used in our analysis and other phenotypes. SNPs related to confounding factors, such as tobacco smoking, body mass index, family history of lung cancer, occupational exposures (asbestos, silica, radon, and heavy metals), and air pollution, were excluded (45). Subsequently, power calculations were carried out using a web tool at https://shiny.cnsgenomics.com/mRnd/ (46). We used the *F* statistics to assess the weak instrumental bias of IVs. When the *F*-statistic was > 10, IVs were considered to be strongly associated with diet-derived antioxidants (47).

### Outcome data sources

The International Lung Cancer Consortium (ILCCO) was established in 2004 to share data from lung cancer epidemiology studies around the world. We acquired GWAS data from the ILCCO using the MRBase database (https://www.mrbase.org/) (48, 49), which included 27,209 subjects of European ancestry, 11,348 had lung cancer in comparison with 15,861 who did not. Among these lung cancer cases, 3,275 were defined as squamous cell lung cancer, and 3,442 were defined as lung adenocarcinoma. There were no overlapping samples between the exposure and outcome.

### Statistical analysis

#### Two-sample Mendelian randomization analysis

We used the multiplicative random-effects inverse-variance weighted (IVW) method as the primary methodology for MR analysis. As a classical method of MR analysis, IVW meta-analyzed Wald ratio estimates for each SNP on the outcome and provided an accurate estimate of the causal effect when all SNPs are valid instrumental variables (50). Then, we used three different methods [MR–Egger, weighted median (WM), and MR-robust adjusted profile score (MR-RAPS)] to test the association between vitamins and lung cancers. The MR–Egger method can assess whether genetic variants have directional pleiotropy and provide a consistent estimate of the causal effect (51). WM estimator combines data on multiple genetic variants into a single causal estimate and provides a consistent estimate if at least half of the weight is derived from valid IVs (52). We selected a relatively higher significant threshold (*p* < 5 × 10^−6^) for genetic variants, and MR-RAPS was used to estimate MR for potentially weak instruments (53).

#### Sensitivity analyses

In addition, we performed several statistical tests for the heterogeneity analysis and the horizontal pleiotropy analysis. Cochran's *Q* statistic for IVW was calculated to evaluate heterogeneity between different SNPs. MR–Egger intercept and MR pleiotropy residual sum and outlier (MR-PRESSO) analyses were both applied to evaluate horizontal pleiotropy. Meanwhile, MR-PRESSO can provide an adjustment effect after removing the outliers (54). We used the MR Steiger test to explore the potential reverse causal effects of lung cancer on diet-derived antioxidants (55). A leave-one-out analysis was performed to estimate whether a single SNP affected the causal relationship between lung cancer and diet-derived antioxidants.

All statistical analyses were performed in R (version 4.1.2) with the “TwoSampleMR,” “MR-PRESSO,” and “mr-raps” packages. We used the Bonferroni correction for multiple testing. The *p*-values <0.004 (0.05/12) were deemed to be strongly associated, and *p*-values > 0.004 and <0.05 were deemed to be suggestively associated. The *p*-values were all two-sided.

### Comparison with meta-analysis

We searched PubMed, Embase, the Cochrane database, and Web of Science to summarize meta-analyses to provide a comprehensive comparison with our MR findings. In the Supplementary Table 5, we describe the results of the meta-analysis in detail.

## Results

### Genetic instruments for diet-derived antioxidants

The characteristics of each participating study are shown in Table 1. Finally, we extracted 48 SNPs associated with diet-derived antioxidants as IVs (Supplementary Table 1). There were no SNPs related to the confounders for lung cancer, such as tobacco smoking, body mass index, family history of lung cancer, etc. (Supplementary Table 2). The average *F* statistic of diet-derived antioxidants ranged from 23 to 24, suggesting a low predisposition for weak instrument bias. In addition, we calculated the statistical power for our MR analyses and found that a minimum detectable odds ratio (OR) ranged from 0.606 to 2.162 when taking into account a type I error of 5% and a statistical power of 0.80 (Supplementary Table 3).

**Table 1:** Characteristics of antioxidant vitamins and lung cancer datasets.

**Exposures**	**Consortium**	**Ancestry**	***F-*statistic**	**Sample size**	**Numbers of SNP**	***R* ^2^**	**Unit of intake**
Retinol	MRC-IEU	European	23	62,991	9	0.00329	μg
Carotene	MRC-IEU	European	23	64,979	16	0.00555	μg
Vitamin C	MRC-IEU	European	23	64,979	11	0.00391	mg
Vitamin E	MRC-IEU	European	24	64,979	12	0.00435	mg
**Outcomes**	**Consortium**	**Ancestry**	**Cases/controls**	**Sample size**			
Lung cancer overall	ILCCO	European	11,348/15,861	27,209			
Squamous cell lung cancer	ILCCO	European	3,275/15,038	18,313			
Lung adenocarcinoma	ILCCO	European	3,442/14,894	18,336			

MRC-IEU, MRC Integrative Epidemiology Unit; ILCCO, International Lung Cancer Consortium; F-statistic, the average of all single-nucleotide polymorphisms.

The value of R^2^ is the sum of the R^2^ values of each SNP.

### Causal effects of diary antioxidant vitamin intake on lung cancer

**Figure 1:**
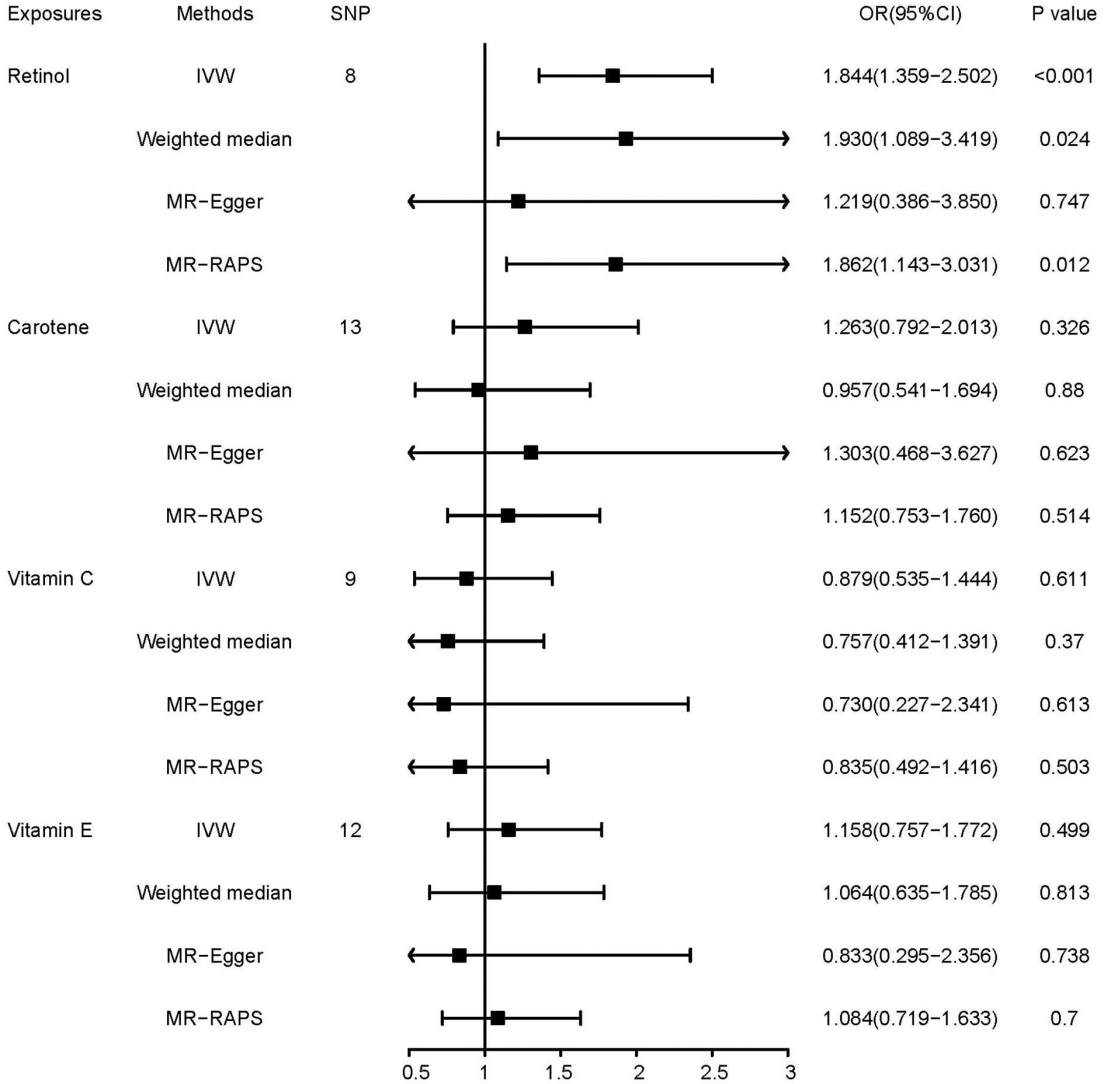
Mendelian randomization (MR) estimates of the associations of antioxidant vitamins with lung cancer.

Of the four antioxidant vitamins, only retinol was found to be causally associated with lung cancer (*OR* = 1.844, 95% *CI*, 1.359–2.502, *p* = 0.00009, Figure 1). In addition, MR–Egger regression, weighted median, and MR-RAPS had similar patterns of results, and MR–Egger regression provided a wider confidence interval than the other methods.

In Cochran's *Q*-test, we found no heterogeneity of antioxidant vitamin IVs in the lung cancer GWAS (*p* = 0.873, Table 2). According to the MR–Egger intercept and MR-PRESSO analysis, there is no evidence of horizontal pleiotropy. The results of the MR Steiger test showed no reverse causality in the analyses. The leave-one-out analysis indicated that the causal effects between antioxidant vitamins and lung cancer were not influenced by any single SNP (Supplementary Figure 3).

**Table 2:** The estimations of heterogeneity and horizontal pleiotropy for Mendelian randomization (MR) results.

**Outcomes**	**Exposures**	**IVW**		**Egger intercept**		**MR-PRESSO**	**MR-Steiger**	
		***Q*-statistics**	***P*-value**	**Intercept**	***P*-value**	***P*-value**	**Correct causal direction**	***P*-value**
Lung cancer overall	Retinol	3.127	8.73E-01	1.76E-02	4.71E-01	9.07E-01	True	4.54E-07
	Carotene	16.819	1.57E-01	−1.38E-03	9.48E-01	1.65E-01	True	7.18E-10
	Vitamin C	10.687	2.20E-01	7.93E-03	7.36E-01	2.14E-01	True	4.25E-07
	Vitamin E	14.085	2.28E-01	1.47E-02	5.09E-01	2.53E-01	True	1.61E-09
Squamous cell lung cancer	Retinol	6.818	4.48E-01	−5.72E-03	8.78E-01	4.54E-01	True	1.06E-04
	Carotene	19.369	8.00E-02	−6.69E-04	9.84E-01	9.90E-02	True	3.33E-05
	Vitamin C	18.062	2.08E-02	4.34E-02	2.89E-01	2.40E-02	True	6.15E-04
	Vitamin E	6.754	8.19E-01	2.06E-02	4.78E-01	8.43E-01	True	1.46E-08
Lung adenocarcinoma	Retinol	2.921	8.92E-01	3.15E-02	4.03E-01	8.92E-01	True	3.89E-06
	Carotene	6.018	9.15E-01	−6.64E-03	7.91E-01	9.27E-01	True	1.98E-09
	Vitamin C	18.005	2.12E-02	5.55E-03	9.12E-01	2.90E-02	True	1.05E-03
	Vitamin E	18.881	6.33E-02	1.69E-02	6.84E-01	6.90E-02	True	1.21E-04

IVW, inverse-variance weighted method; MR-PRESSO, MR pleiotropy residual sum and outlier.

### Causal effects of dietary antioxidant vitamin intake on squamous cell lung cancer

**Figure 2:**
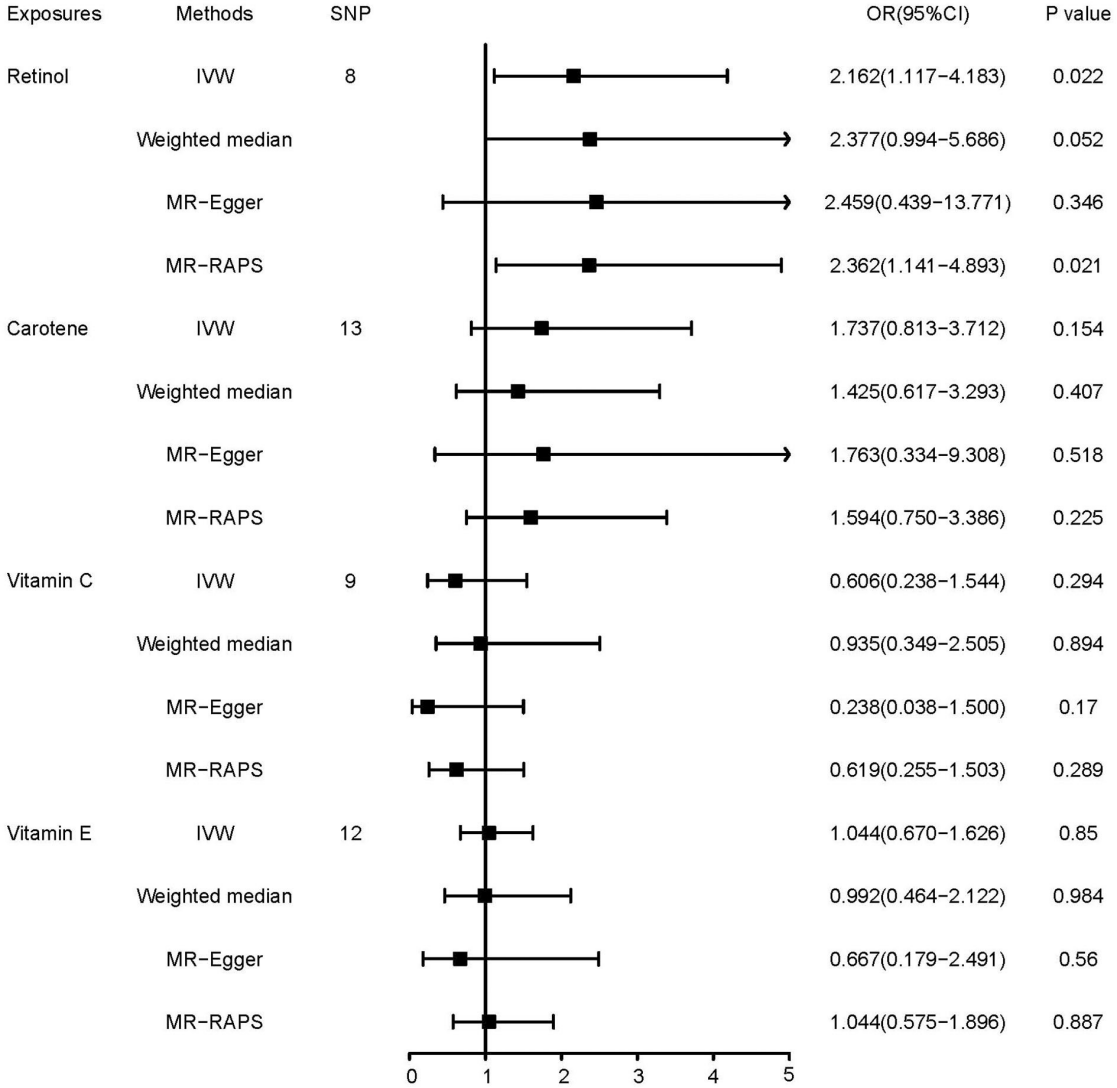
Mendelian randomization estimates of the associations of antioxidant vitamins with squamous cell lung cancer.

A suggestive causal effect of dietary retinol intake on the risk of squamous cell lung cancer was found in this two-sample MR analysis (*OR* = 2.162, 95% *CI*, 1.117–4.183, *p* = 0.022, Figure 2). MR-RAPS obtained the same result. However, we observed no significant relationship with other antioxidant vitamins.

We did not find any heterogeneity or horizontal pleiotropy in the analyses except for the causal effect of vitamin C on squamous cell lung cancer (*p* for Cochran's *Q*-test = 0.021, *p* for MR-PRESSO = 0.024, Table 2). In this MR-PRESSO analysis, rs74978963 was regarded as the outlier. After correcting for the outlier, we obtained a consistent conclusion with the raw analysis (Supplementary Table 4). Based on the MR Steiger test, we did not find any reverse causality.

### Causal effects of dietary antioxidant vitamin intake on lung adenocarcinoma

**Figure 3:**
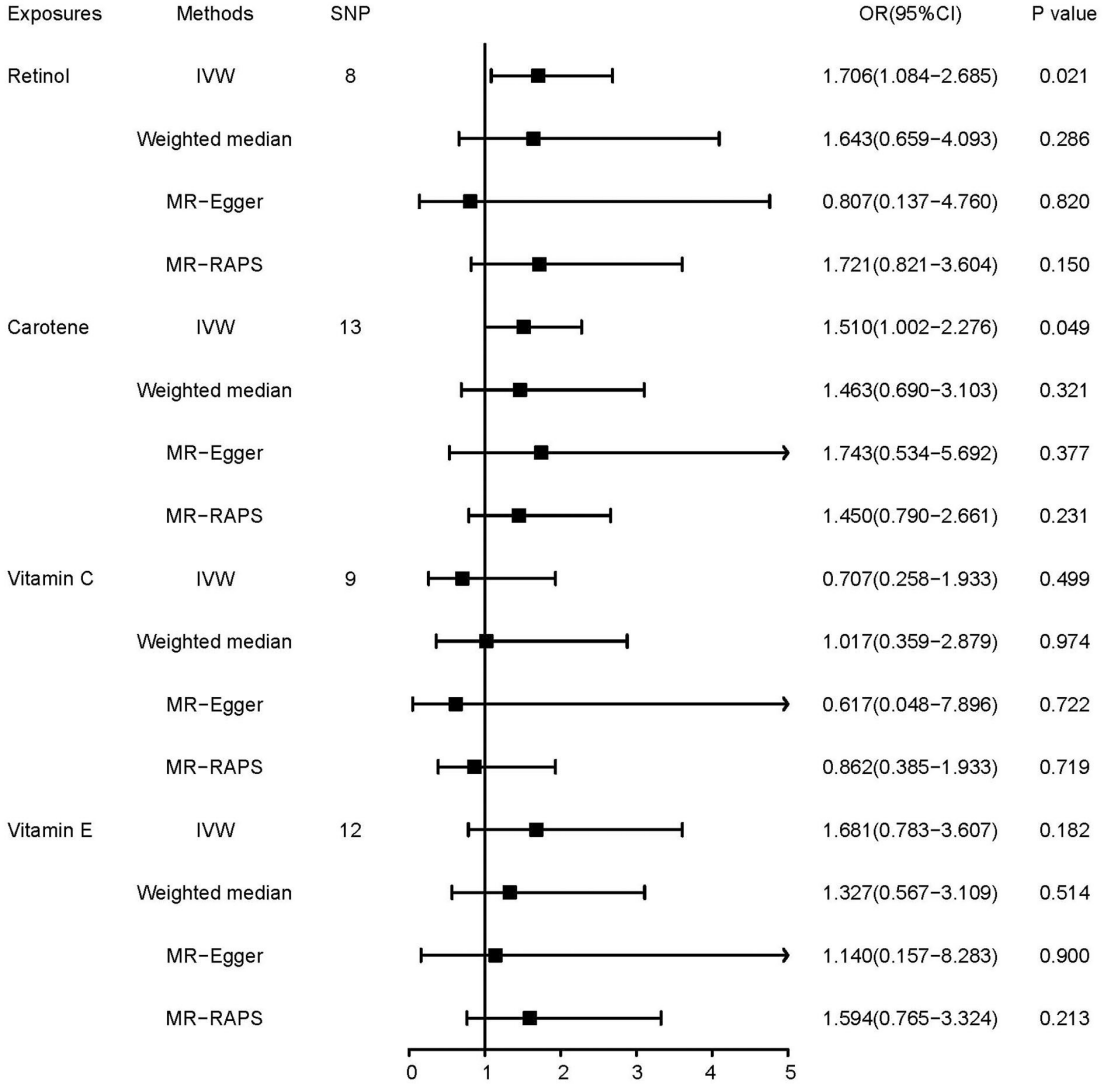
Mendelian randomization estimates of the associations of antioxidant vitamins with lung adenocarcinoma.

We found a suggestive causal effect of dietary retinol intake (*OR* = 1.706, 95% *CI*, 1.084–2.685, *p* = 0.021, Figure 3) and dietary carotene intake (*OR* = 1.510, 95% *CI*, 1.002–2.276, *p* = 0.049, Figure 3) on the risk of lung adenocarcinoma. However, the association was not significant between the intake of other dietary antioxidant vitamins and lung adenocarcinoma.

Both heterogeneity and horizontal pleiotropy were found in the analyses of dietary vitamin C intake on the risk of lung adenocarcinoma (*p* for Cochran's *Q*-test = 0.021 and *p* for MR-PRESSO = 0.029, Table 2). MR-PRESSO analysis detected rs114598078 as an outlier, and we concluded the same result after correcting for the outlier (Supplementary Table 3). In the MR Steiger test, we found no reverse causal effects of lung cancer on the dietary antioxidant and vitamin intake.

### Meta-analysis between antioxidant vitamins and risk of lung cancer

After our search, a total of eight meta-analyses were included in the study (Supplementary Table 5) (25–27, 37, 56–59), of which four meta-analyses included vitamin A (25, 56, 58, 59), four included vitamin C (26, 37, 58, 59), four included vitamin E (27, 57–59), and only one included carotene (25). The results of all meta-analyses are not completely consistent. For vitamin A, only one meta-analysis showed that vitamin A can reduce the incidence of lung cancer (25), while for vitamin C, three meta-analyses showed that it can reduce the incidence of lung cancer (26, 37, 58). For carotene and vitamin E, only one article showed that they can reduce the incidence of lung cancer (25, 27).

## Discussion

In this Mendelian randomization analysis, we used summary data from GWASs. Four diet-derived antioxidant vitamins were selected as exposures, and lung cancer and its common pathological types were selected as outcomes. The intake of dietary retinol was strongly associated with lung cancer. Furthermore, the suggestive causal effects of dietary retinol intake on the risk of squamous cell cancer and lung adenocarcinoma were also found in this MR analysis. Additionally, dietary carotene intake was suggestively correlated with lung adenocarcinoma. We could not find significant associations between other dietary antioxidant vitamins (vitamin C and vitamin E) and lung cancer and its subtypes.

Previous studies have shown that the occurrence of lung cancer is related to genetic and environmental factors, such as tobacco, alcohol, asbestos, and other chemical products (42). Oxidative stress in the respiratory system increases the production of pulmonary inflammation mediators and initiates or promotes the mechanisms of carcinogenesis (60). Dietary antioxidant vitamins, such as retinol, carotene, vitamin C, and vitamin E, which are primarily obtained through vegetables, fruits, and grains (61), were hypothesized to decrease the lung cancer risk (62). Based on the above results and hypothesis, in recent years, some studies have begun to focus on whether antioxidant vitamins can prevent lung cancer (63, 64). However, the correlation between antioxidant vitamins and lung cancer is still controversial (65, 66). In addition, we reviewed the previously published meta-analysis and found that the conclusions regarding antioxidant vitamins in the protection against lung cancer are not consistent, which may be due to the different inclusion criteria of each meta-analysis, as well as the different types of studies and sample sizes. MR studies can reduce the impact of these factors and draw more credible conclusions.

Our results showed that a higher dietary retinol intake can promote the development of lung cancer, which is consistent with the serum retinol results (*OR* = 1.44, 95% *CI*, 1.01–2.06, *p* = 0.04) (42). The statistical power of MR analysis was more than 80%, increasing the reliability of our results. Narita et al. performed a prospective cohort study in Japan and found that the higher consumption of the dietary antioxidant retinol was positively associated with lung cancer risk in men, especially in current smokers (28). The multicenter, randomized, double-blind, placebo-controlled primary prevention trial, the Beta Carotene and Retinol Efficacy Trial, also showed that the incidence rate of the experimental group with more retinol supplements was higher than that of the control group (67). Satia et al. proposed that long-term use of retinol supplements was associated with a higher risk for lung cancer overall [hazard ratios (*HR*s) = 1.53, 95% *CI*, 1.12–2.08] and non-small cell lung cancer (*HR*s = 1.80, 95% *CI*, 1.29–2.52), according to a cohort study (68). These results were in accordance with this MR analysis. Several putative mechanisms may explain the carcinogenic effects of retinol. Some researchers found that carotenoids or micronutrients had significant protective effects against carcinogenesis. However, higher retinol intake may interfere with the absorption, transport, distribution, and metabolism of carotenoids and micronutrients, which increases the risk of lung cancer (56, 68). Previous studies suggest that retinol may enhance free radical production, leading to phosphorylation of Src-tyrosine kinase, MAPK/ERK kinases 1/2 (MEK1/2), cAMP-responsive element binding protein (CREB), and extracellular signal-regulated kinases 1 and 2 (ERK1/2). The activation of this pathway appears to be involved in the onset of some of the deleterious effects, such as malignant transformation and cell proliferation (69, 70).

Regarding the relationship between carotene and lung cancer, our study showed that a higher dietary intake of carotene can suggestively increase the risk of lung adenocarcinoma, but this conclusion was not observed in lung cancer overall or squamous cell carcinoma. However, a prospective cohort study showed that the increased intake of foods containing carotenoids can decrease the risk of lung cancer, but that study did not evaluate subtypes of carotenoids, such as β-carotene (71). In contrast, a placebo-controlled, randomized intervention trial, the Alpha-Tocopherol Beta-Carotene (ATBC) Cancer Prevention Study, found that smokers supplemented with beta-carotene had a higher risk of lung cancer (72). Similarly, Middha et al. conducted a randomized, double-blind intervention trial that showed that beta-carotene supplementation increased the risk of lung cancer in smokers regardless of the tar or nicotine content of the cigarettes smoked (69). In a ferret model that was given β-carotene supplements and exposed to cigarette smoke for 6 months, the results showed that β-carotene inhibited retinoid signaling by decreasing the RARβ gene expression and amplifying the expression of activator protein-1, thereby promoting cancer formation (73). Of note, the inconsistent results of different lung cancer pathotypes in this MR analysis suggested that different pathotypes may have different responses to carotene. Further studies are necessary to explore the mechanisms of specific types of carotene on the risk of lung cancer in different pathological types.

Some studies have shown that the intake of vitamin C has no effect on the occurrence of lung cancer (28, 59). A population-based prospective study revealed that the dietary consumption of vitamin C was not associated with overall lung cancer (28). Furthermore, our results suggested a non-significant association between lung cancer and vitamin C, which is one of the most common antioxidants found in vegetables and fruits (65), and has generally been acknowledged to protect cells from oxidative DNA damage, thus exerting chemopreventive effects and blocking carcinogenesis (74). Several observational studies found that dietary vitamin C intake from food sources showed a significant protective effect on lung cancer (71, 75, 76). These controversial results might be because participants who consumed high amounts of dietary vitamin C were more health-conscious (77).

Our results did not find a causal effect of dietary vitamin E intake on lung cancer. There is no consensus on the relationship between vitamin E and lung cancer risk (78–80). An observational prospective study suggested that healthy people who self-select for more vitamin E through diet or supplements had a lower risk of developing lung cancer (18). In addition, a prospective study called the Shanghai Women's Health Study (SWHS) revealed a protective association between tocopherol intake and lung cancer in women exposed to side-stream smoke; conversely, vitamin E supplements can increase lung cancer risk overall (*HR* = 1.33, 95% *CI*, 1.01–1.73) and lung adenocarcinoma risk (*HR* = 1.79, 95% *CI*, 1.23–2.60) (78). However, in the Netherlands Cohort Study on Diet and Cancer, which was a prospective cohort study, vitamin E was not found to be related to the risk of lung cancer incidence (79). A post-intervention follow-up study also revealed a non-significant correlation between supplementary vitamin E and lung cancer (80). More experiments are required to verify the corresponding relationship.

The strength of our article was the use of MR analysis. Summary data with a large sample size were used, and the confounding and reverse causality, which are prevalent in conventional observational studies, were reduced. More importantly, MR analysis can be implemented at any point without time and resource requirements, which are necessary for randomized controlled trials. Thus, MR analysis reduces the likelihood that subjects will be exposed to unnecessary risk and harm. The participants enrolled in this study were of European ancestry, which reduces the effect of ethnic differences. Furthermore, Cochran's *Q*-test, MR–Egger, MR-PRESSO, and Steiger filtering methods for the sensitivity analysis validated that our findings were unlikely to be affected by pleiotropic effects or reverse causality.

However, this study has several limitations. First, we used *p* < 5 × 10^−6^ as the threshold for IV selection, which may lead to weak instrumental bias. However, the *F*-statistic for each IV was more than 10, and the results of MR-RAPS showed that weak IVs could not reduce the credibility of our results. Second, we were unable to determine the non-linear causal association between antioxidant vitamin intake and lung cancer risk, and due to the lack of SNPs corresponding to the specific type of carotene, we could not clarify the specific role of each specific type of carotene. The role of antioxidant vitamins in small cell lung cancer has not been studied due to the lack of SNPs. Third, the genetic variants were restricted to European samples, which may prevent the generalization of our results to other populations. Finally, the statistical power of these MR analyses may be limited. This may be due to the low variability of antioxidant vitamin intake explained by IVs and the low proportion of cases. Thus, the results with low statistical power should be interpreted carefully.

## Conclusion

In summary, this MR analysis showed that a higher dietary retinol intake could increase the lung cancer risk. Dietary retinol intake may have a causal effect on squamous cell cancer and lung adenocarcinoma. In addition, the higher dietary carotene intake may also increase the risk of lung adenocarcinoma. Our MR analysis does not support the protective role of dietary antioxidant vitamin intake on the risk of lung cancer development, which delivers an important public dietary and healthy message that administration of antioxidant vitamins may not be necessary for the prevention of lung cancer and provides a basis for future research. Nevertheless, by performing further large MR studies and clinical trials, the relationship between vitamins and lung cancer may be clarified, particularly the relationship between specific vitamin types and more lung cancer subtypes.

## Data availability statement

The original contributions presented in the study are included in the article/Supplementary material, further inquiries can be directed to the corresponding author/s.

## Author contributions

HZ conceived of the presented idea. XJ developed the theory and performed the computations. HZ and XJ verified the analytical methods. All authors discussed the results and contributed to the final manuscript.

## Conflict of interest

The authors declare that the research was conducted in the absence of any commercial or financial relationships that could be construed as a potential conflict of interest.

## Publisher's note

All claims expressed in this article are solely those of the authors and do not necessarily represent those of their affiliated organizations, or those of the publisher, the editors and the reviewers. Any product that may be evaluated in this article, or claim that may be made by its manufacturer, is not guaranteed or endorsed by the publisher.
