# Supplementary material for: Causal associations between dietary antioxidant vitamin intake and lung cancer: A Mendelian randomization study
Source: Front Nutr. 2022 Sep 2;9:965911. doi: 10.3389/fnut.2022.965911 (PMC9479338; doi:10.3389/fnut.2022.965911)
Supplement: Supplementary file 1 [file Data_Sheet_1.pdf]

Table S1. Single nucleotide polymorphisms (SNPs) associated with diet-derived antioxidants

SNP	Exposure	Effect allele	Other allele	EAF	*P* value	SE	Beta	R^2	*F*-statistic
rs12119164	Retinol	G	A	0.742193	3.40E-06	0.006371	0.0295741	0.00034	22
rs692790	Retinol	C	T	0.874331	1.40E-06	0.008382	0.0404149	0.00037	23
rs74977546	Retinol	A	G	0.052896	5.50E-07	0.012678	-0.063514	0.00040	25
rs149577802	Retinol	T	C	0.015116	3.40E-06	0.023412	-0.108795	0.00034	22
rs3213829	Retinol	G	T	0.546482	3.60E-06	0.00561	0.025976	0.00034	21
rs117669768	Retinol	A	G	0.038236	9.00E-08	0.014768	0.0789545	0.00045	29
rs2126371	Retinol	T	C	0.317531	1.30E-06	0.005977	-0.0289453	0.00037	23
rs117219913	Retinol	C	T	0.07834	4.40E-06	0.010381	0.0476864	0.00033	21
rs909570	Retinol	A	G	0.938952	4.40E-06	0.011572	-0.053135	0.00033	21
rs1936052	Carotene	T	C	0.155936	2.50E-06	0.007668	-0.0361185	0.00034	22
rs6660246	Carotene	C	A	0.450266	1.40E-06	0.005531	-0.0266889	0.00036	23
rs12126792	Carotene	G	A	0.011509	1.50E-06	0.028005	-0.134712	0.00036	23
rs77547747	Carotene	C	T	0.056418	2.40E-06	0.011834	-0.055803	0.00034	22
rs6596473	Carotene	C	G	0.299056	3.70E-06	0.005976	0.0276423	0.00033	21
rs62417408	Carotene	G	A	0.037772	2.40E-06	0.014655	-0.0691153	0.00034	22
rs16898247	Carotene	A	G	0.018968	9.00E-08	0.020031	-0.107091	0.00044	29
rs13295574	Carotene	A	G	0.303383	3.70E-06	0.005982	-0.0276722	0.00033	21
rs3829931	Carotene	A	T	0.973235	3.00E-06	0.01779	0.0830829	0.00034	22
rs17800766	Carotene	C	T	0.011673	1.80E-06	0.025496	-0.121685	0.00035	23
rs2998143	Carotene	G	A	0.603581	1.80E-06	0.00584	-0.0278722	0.00035	23
rs4771831	Carotene	A	G	0.351886	4.20E-06	0.005762	-0.0265212	0.00033	21
rs116995905	Carotene	T	C	0.009812	4.40E-06	0.028806	-0.132301	0.00032	21
rs366337	Carotene	G	A	0.936764	1.30E-06	0.011222	0.0543804	0.00036	23
rs117731008	Carotene	A	G	0.016818	4.20E-06	0.021228	0.0977133	0.00033	21

rs5760695	Carotene	C	T	0.086598	3.20E-06	0.010056	0.0468481	0.00033	22
rs7626478	Vitamin C	A	G	0.720257	4.50E-06	0.006101	0.0279796	0.00032	21
rs114598078	Vitamin C	T	C	0.042309	1.90E-06	0.013764	0.0655782	0.00035	23
rs4481190	Vitamin C	C	A	0.351041	9.60E-08	0.005744	-0.0306375	0.00044	28
rs74978963	Vitamin C	T	C	0.008729	1.20E-06	0.031021	0.150814	0.00036	24
rs61868302	Vitamin C	T	C	0.060676	1.40E-06	0.011839	-0.0571013	0.00036	23
rs17482258	Vitamin C	T	C	0.099073	3.70E-06	0.00926	0.042829	0.00033	21
rs2018201	Vitamin C	G	T	0.026551	2.50E-06	0.017165	-0.0808079	0.00034	22
rs9540734	Vitamin C	A	G	0.477524	2.30E-06	0.005485	-0.0259285	0.00034	22
rs4238567	Vitamin C	C	T	0.522046	4.30E-06	0.005508	0.0253068	0.00032	21
rs11650824	Vitamin C	A	T	0.035071	5.60E-07	0.015882	0.0794787	0.00039	25
rs1883993	Vitamin C	A	G	0.095419	1.50E-06	0.009353	0.044959	0.00036	23
rs536912	Vitamin E	A	C	0.736049	9.00E-07	0.006201	0.0304596	0.00037	24
rs6033	Vitamin E	G	A	0.072493	9.90E-07	0.010556	-0.0516563	0.00037	24
rs2723979	Vitamin E	G	T	0.583758	1.50E-06	0.005527	-0.0266142	0.00036	23
rs979218	Vitamin E	C	A	0.098036	3.10E-06	0.009223	-0.0430325	0.00033	22
rs79966958	Vitamin E	T	C	0.012689	2.00E-06	0.024505	-0.116527	0.00035	23
rs12421920	Vitamin E	G	A	0.093546	3.70E-06	0.009385	-0.0434479	0.00033	21
rs111306778	Vitamin E	A	G	0.089621	5.40E-07	0.009572	-0.0479658	0.00039	25
rs4903544	Vitamin E	T	C	0.30035	9.50E-07	0.006021	-0.0295098	0.00037	24
rs12899673	Vitamin E	A	G	0.333079	3.80E-06	0.005818	0.0268951	0.00033	21
rs35218694	Vitamin E	G	A	0.034459	1.30E-06	0.01531	-0.0741637	0.00036	23
rs71385328	Vitamin E	G	A	0.011307	7.00E-07	0.026206	0.130015	0.00038	25
rs12165526	Vitamin E	A	T	0.100636	1.80E-07	0.009178	0.0479017	0.00042	27

Note: SNP: Single nucleotide polymorphisms; EAF: effect allele frequency; SE, standard error; R^2 was calculated as follows: $2*beta^2*EAF*(1-EAF)/(2*beta2*EAF*(1-EAF) + se^2*2*N*EAF(1-EAF))$. The *F*-statistic for each SNP was calculated as follows: $F = (N - 2) *R^2/ (1 - R^2)$.

Table S2: instrumental variable trait of dietary antioxidants in PhenoScanner V2.

rsID	Trait	PMID	Beta	*P* value	Sample size
rs692790	Immature fraction of reticulocytes	27863252	0.03421	1.08E-10	173480
rs117669768	Unspecified nephritic syndrome	UKBB	0.000417	9.68E-06	337199
rs6596473	Overall health rating	UKBB	-0.01014	1.63E-07	336020
rs6596473	Height	UKBB	0.009607	3.97E-07	336474
rs6596473	Time spent watching television	UKBB	-0.01002	1.04E-06	319740
rs6596473	Trunk fat-free mass	UKBB	0.007806	3.37E-06	331030
rs6596473	Trunk predicted mass	UKBB	0.007775	3.43E-06	330995
rs6596473	Sitting height	UKBB	0.009305	6.35E-06	336172
rs6596473	Pulse rate	UKBB	-0.01221	8.78E-06	317756
rs17800766	Treatment with nitrofurantoin	UKBB	0.001206	6.36E-06	337159
rs4771831	Height	UKBB	-0.01002	3.37E-08	336474
rs4771831	Height	25282103	-0.017	2.00E-07	247997
rs4771831	Height	23754948	-0.02528	2.03E-07	129315
rs4771831	Comparative height size at age 10	UKBB	-0.00893	3.09E-07	332021
rs4771831	Height	20881960	NA	9.69E-07	129476
rs4771831	Height	20881960	NA	9.69E-07	133653
rs116995905	Home area population density: Scotland - remote rural	UKBB	-0.00045	1.01E-10	333997
rs116995905	Cause of death: peripheral vascular disease, unspecified	UKBB	-0.01222	2.80E-09	7637
rs116995905	Cause of death: hodgkins disease, unspecified	UKBB	-0.01202	9.44E-08	7637
rs116995905	Treatment with amisulpride	UKBB	-0.00091	1.54E-07	337159
rs74978963	Cause of death: pneumonitis due to food and vomit	UKBB	-0.01381	9.54E-07	7637
rs9540734	Frequency of unenthusiasm or disinterest in last 2 weeks	UKBB	0.007025	1.64E-06	326730
rs9540734	Breastfed as a baby	UKBB	-0.00593	2.80E-06	255881

rs9540734	Medication for pain relief, constipation, heartburn: none of the above	UKBB	-0.00571	2.86E-06	333581
rs9540734	Miserableness	UKBB	0.005411	7.42E-06	331856
rs9540734	Neuroticism score	UKBB	0.03863	9.41E-06	274108
rs6033	Blood protein levels	28240269	-0.8454	5.00E-25	-
rs979218	Treatment with epaderm ointment	UKBB	0.000458	5.02E-06	337159
rs79966958	Cause of death: pharynx, unspecified	UKBB	-0.01028	5.96E-06	7637
rs12899673	Time spent watching television	UKBB	-0.00907	5.05E-06	319740
rs12899673	Waist circumference	UKBB	-0.01032	6.46E-06	336639
rs71385328	Cause of death: cerebrum, except lobes and ventricles	UKBB	-0.01052	5.95E-07	7637
rs12165526	Cause of death: cholangitis	UKBB	0.005036	2.49E-07	7637

Note: UKBB: UK biobank

Table S3. Power calculation for the two-sample Mendelian randomization analyses of antioxidant vitamins on lung cancer.

Outcomes	Exposures	Required ORs (power = 80%)	Estimated ORs
Lung cancer overall	Retinol	1.755	1.844
	Carotene	1.552	1.263
	Vitamin C	0.563	0.879
	Vitamin E	1.636	1.158
Squamous cell lung cancer	Retinol	2.050	2.162
	Carotene	1.794	1.737
	Vitamin C	0.288	0.606
	Vitamin E	1.902	1.044

Lung adenocarcinoma	Retinol	2.035	1.706
	Carotene	1.782	1.510
	Vitamin C	0.304	0.707
	Vitamin E	1.889	1.681

Note: OR: odds ratio

Table S4. MR-PRESSO outlier-corrected MR analysis for antioxidant vitamin intake and lung cancer.

Outcomes	Exposures	Outliers	Raw OR (95% CI)	*P* value	Corrected OR (95% CI)	*P* value	Global test	Distortion Test
Squamous cell lung cancer	Vitamin C	rs74978963	0.606(0.238-1.544)	0.325	1.079(0.460-2.529)	0.867	0.024	0.033
Lung adenocarcinoma	Vitamin C	rs114598078	0.707(0.258-1.933)	0.518	1.182(0.537-2.601)	0.690	0.029	0.106

Table S5. Characteristics of the included meta-analysis of the association between antioxidant vitamin intakes and the lung cancer outcomes.

First author	Publication years	Publication country	Exposures	Outcomes	Risk estimates (RR/HR/OR) and 95% CIs	*P* value
Fritz et al[1]	2011	Canada	Vitamin A	Lung cancer	The risk was not estimated due to heterogeneity in the reporting of results.	Not mentioned
Luo et al[2]	2014	China	Vitamin C	Lung cancer	RR=0.93, 95%CI=0.88-0.98	P<0.05
Zhu et al[3]	2017	China	Vitamin E	Lung cancer	RR=0.95, 95% CI =0.91-0.99	P<0.05
Yu et al[4]	2015	China	Vitamin A and β-Carotene	Lung cancer	RR=0.855, 95%CI=0.739-0.989 for vitamin A; RR=0.768, 95%CI=0.675-0.874 for β-Carotene	P=0.005 for vitamin A; P=0.002 for β-carotene
Alkhenizan et al[5]	2007	Saudi Arabia	Vitamin E	Lung cancer	RR=1.02, 95%CI=0.88-1.19	P>0.05
Cho et al[6]	2006	USA	Vitamin A, C and E	Lung cancer	RR=0.96, 95%CI=0.83-1.11 for vitamin A; RR=0.80, 95% CI=0.71-0.91 for vitamin C; RR=0.86, 95% CI=0.76-0.99 for vitamin E	P=0.90 for vitamin A; P=0.002 for vitamin C; P=0.36 for vitamin E
Fu et al[7]	2021	China	Vitamin C	Lung cancer	RR=0.84, 95%CI=0.73-0.97	P<0.05
Cortés-Jofré et al[8]	2020	Chile	Vitamin A, C and E	Lung cancer	RR=1.09, 95%CI=1.00-1.19 for vitamin A; RR=1.29, 95% CI=0.67-2.49 for vitamin C; RR=1.01, 95% CI=0.90-1.14 for vitamin E	P>0.05 for all vitamins

Supplemental Figure 1 Flowchart of Mendelian randomization framework in this study.

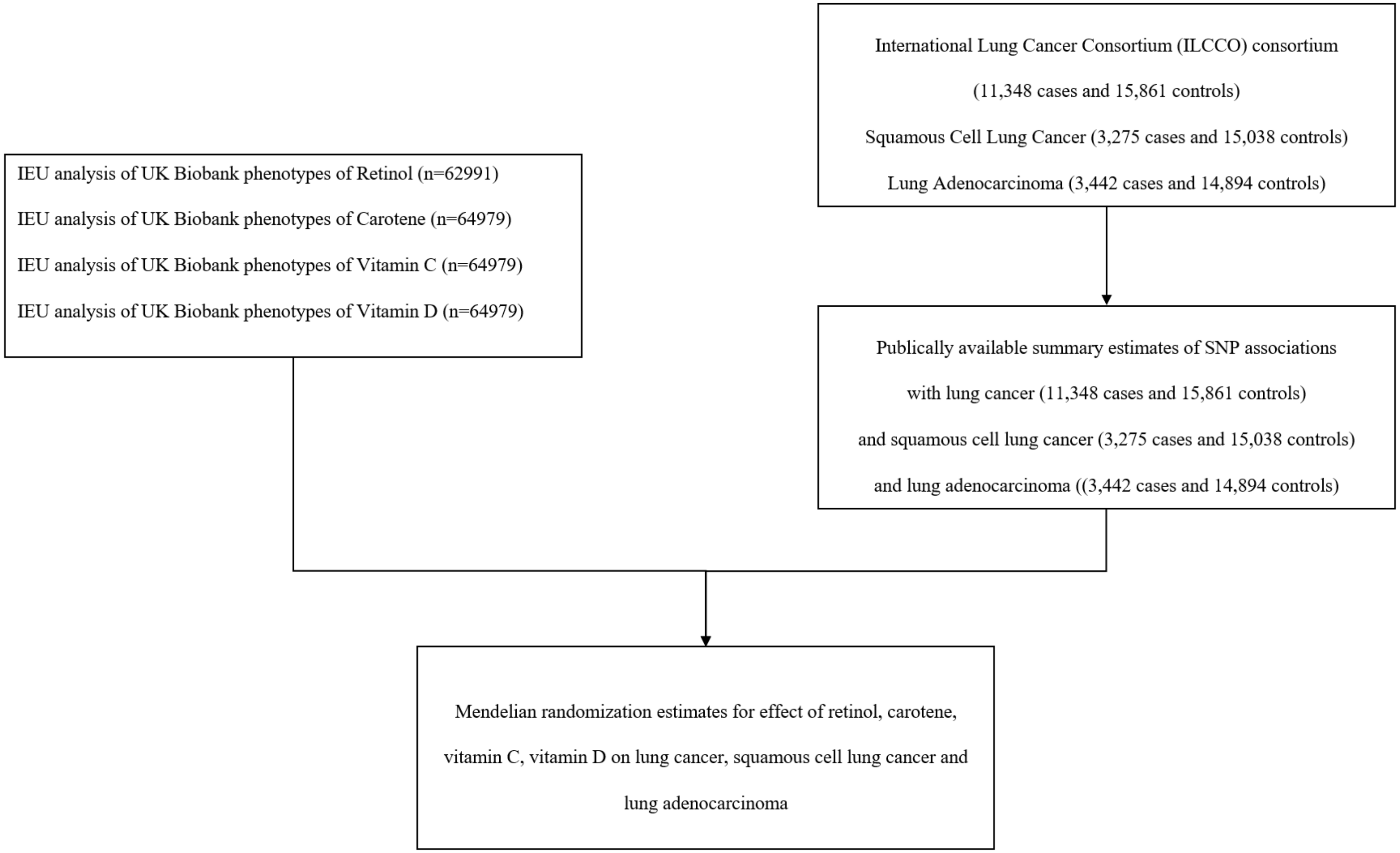

Note: IEU: Integrative Epidemiology Unit

Supplemental Figure 2 Diagram of Mendelian randomization framework in this study.

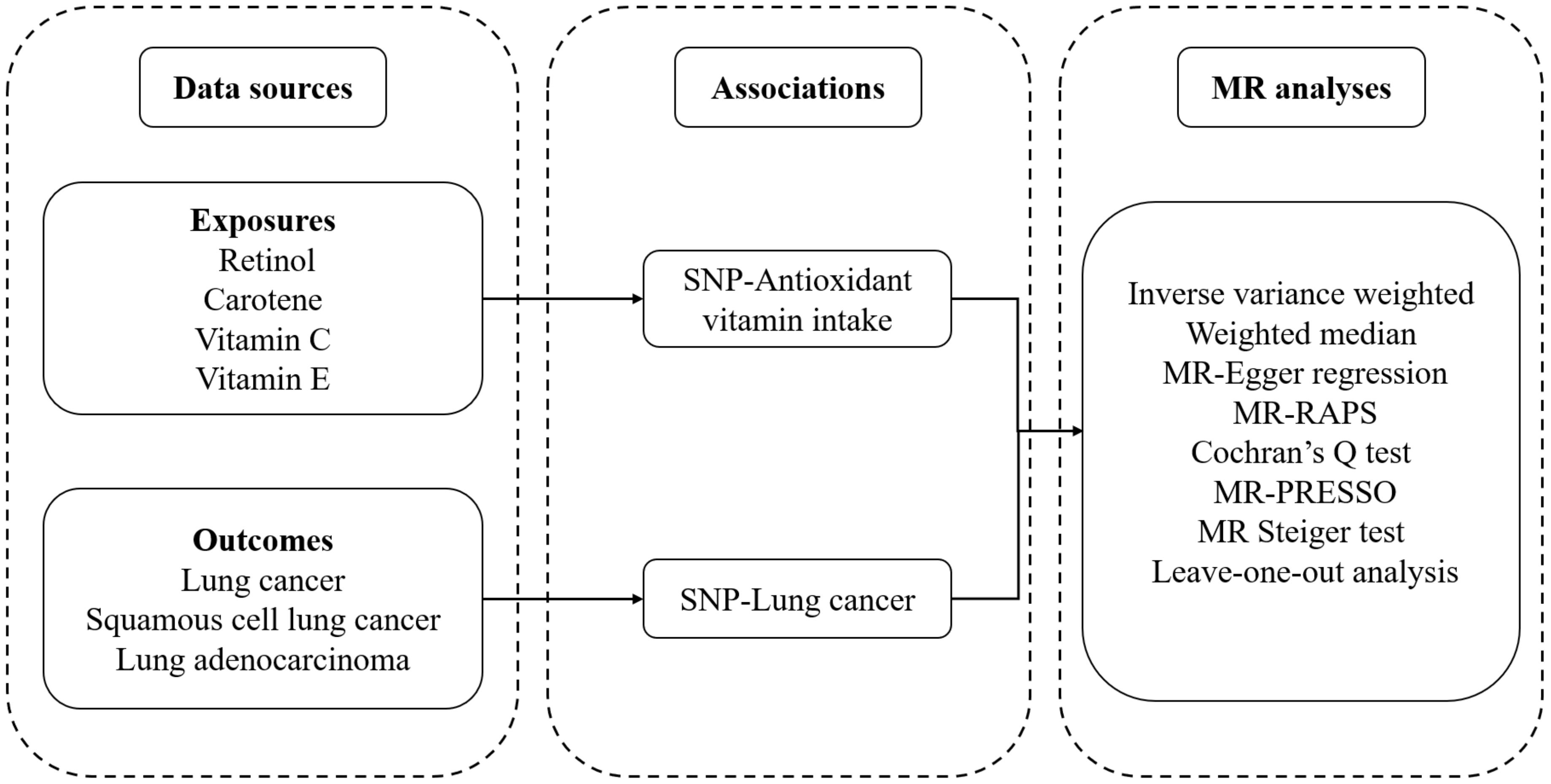

Supplemental Figure 3 Leave-one-out test for genetically determined antioxidant vitamins and risk of lung cancer (A, B, C, D); squamous cell lung cancer (E, F, G, H); lung adenocarcinoma (I, J, K, L)

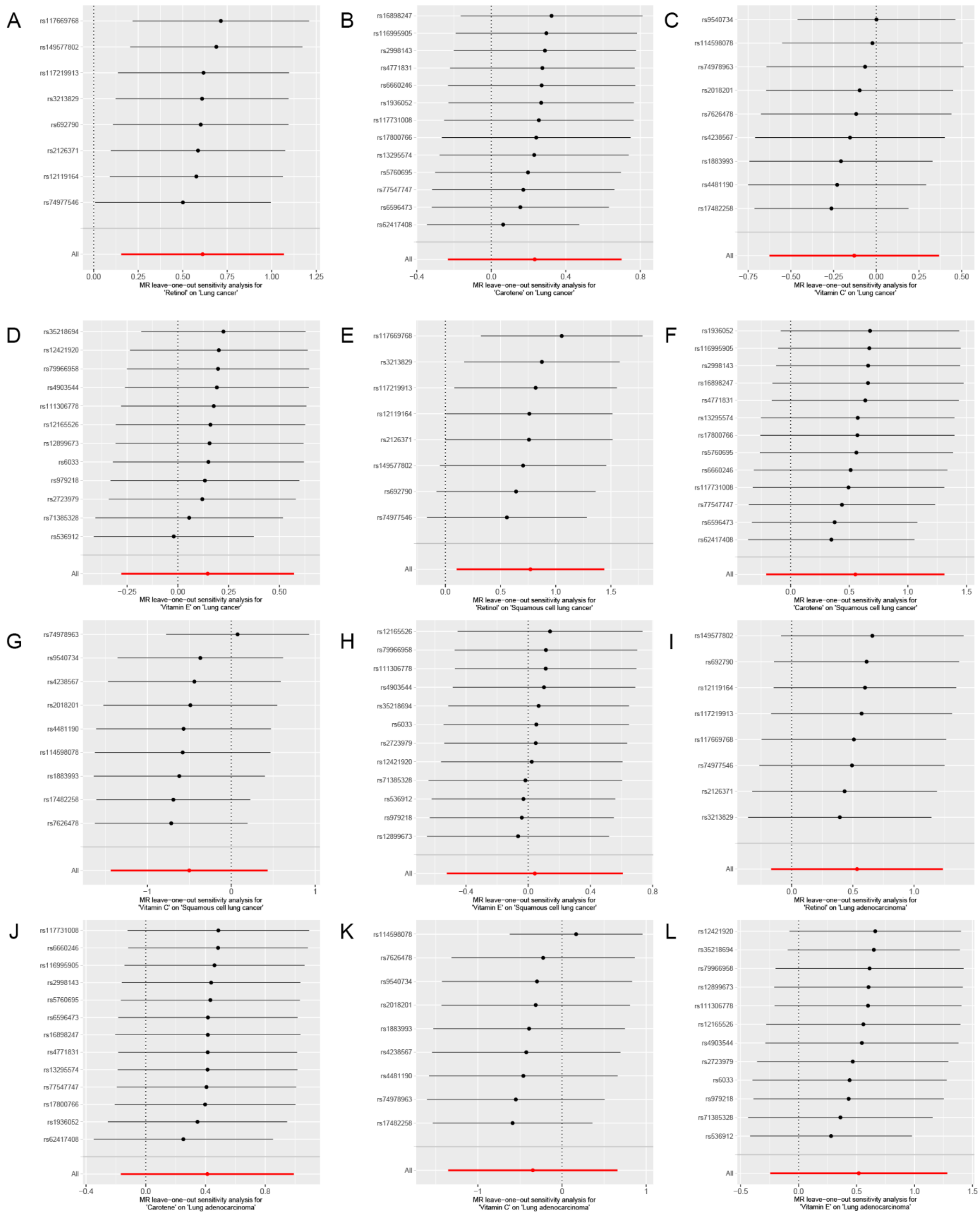

Reference

[1] Fritz H, Kennedy D, Fergusson D, et al. Vitamin A and retinoid derivatives for lung cancer: a systematic review and meta analysis[J]. PLoS One, 2011, 6(6): e21107.
[2] Luo J, Shen L, Zheng D. Association between vitamin C intake and lung cancer: a dose-response meta-analysis[J]. Sci Rep, 2014, 4: 6161.
[3] Zhu Y J, Bo Y C, Liu X X, et al. Association of dietary vitamin E intake with risk of lung cancer: a dose-response meta-analysis[J]. Asia Pac J Clin Nutr, 2017, 26(2): 271-277.
[4] Yu N, Su X, Wang Z, et al. Association of Dietary Vitamin A and β-Carotene Intake with the Risk of Lung Cancer: A Meta-Analysis of 19 Publications[J]. Nutrients, 2015, 7(11): 9309-24.
[5] Alkhenizan A, Hafez K. The role of vitamin E in the prevention of cancer: a meta-analysis of randomized controlled trials[J]. Ann Saudi Med, 2007, 27(6): 409-14.
[6] Cho E, Hunter D J, Spiegelman D, et al. Intakes of vitamins A, C and E and folate and multivitamins and lung cancer: a pooled analysis of 8 prospective studies[J]. Int J Cancer, 2006, 118(4): 970-8.
[7] Fu Y, Xu F, Jiang L, et al. Circulating vitamin C concentration and risk of cancers: a Mendelian randomization study[J]. BMC Med, 2021, 19(1): 171.
[8] Cortés-Jofré M, Rueda J R, Asenjo-Lobos C, et al. Drugs for preventing lung cancer in healthy people[J]. Cochrane Database Syst Rev, 2020, 3(3): Cd002141.
